# Thopaz Portable Suction Systems in Thoracic Surgery: An end user assessment and feedback in a tertiary unit

**DOI:** 10.1186/1749-8090-6-59

**Published:** 2011-04-21

**Authors:** Sridhar Rathinam, Amy Bradley, Teresa Cantlin, Pala B Rajesh

**Affiliations:** 1 Regional Department of Thoracic Surgery, Birmingham Heartlands Hospital, Bordesley Green East, Birmingham B9 5SS, UK; 2 Consultant Thoracic Surgeon, University Hospitals of Leicester, Leicester LE3 9QP, UK

## Abstract

**Background:**

Thoracic surgical patients have chest drains inserted to enable re-expansion of lungs, to clear contents from the pleural cavity which sometimes require negative suction. Suction impedes mobility, may have variable suction delivery and increases risk of infection. Assessment of air-leak in conventional drains is not scientific and is subjective. Thopaz chest drain system is a portable suction unit which allows mobilization of the patient, with scientific digital flow recordings and an in built alarm system.

**Methods:**

We evaluated the utility, staff and patient feedback of this device in a pilot evaluation in a regional thoracic unit in a structured format over a period of two months. Staff responses were graded on a scale of 1 to 6 [1 Excellent to 6 Poor].

**Results:**

120 patients who underwent elective bullectomy/pleurectomy, VATS lung biopsies, VATS metastectomy and lung resections were evaluated. The staff feedback forms were positive. The staff liked the system as it was more scientific and accurately recordable. It made nursing and physiotherapy easier as they could mobilise patients early. The patients liked the compact design, weightlessness and the silence. It enabled mobilisation of the patients and scientific removal of chest drain.

**Conclusions:**

Thopaz digital suction units were found to be user friendly and were liked by the staff and patients. The staff feedback stated the devices to be objective and scientific in making decisions about removal and enabled mobilisation.

## Introduction

Thoracic surgical patients have chest drains placed in the pleura to drain air and blood [1]. There are various devices which are connected to the chest drains with some of the recent ones having digital flow meters incorporated in them [2]. In some circumstances these drains are placed on negative pressure suction to evacuate the contents of the pleural cavity as well as to help re-expand the lungs [3]. Traditionally this has been achieved by connecting the chest drain bottles to low pressure wall suction. Though this achieves negative suction this has limitations as it impairs patient mobility and the suction applied by the wall unit can be variable. There is a potential infection risk particularly if patients disconnect themselves to mobilise leaving the suction tube on the floor and reattach to the drain later.

There are currently systems which generate the flows by digital meters incorporated in the drainage portals [4]. However this still doesn't solve the problem of mobilisation of the patient as if the patient needs suction they have to be connected to the wall.

The Thopaz chest drain system (Medela Switzerland) is a portable suction unit with a drainage canister which comes with a mains charger and can be allowed to mobilise with the patient.

We describe the user feedback from staff and patients in our regional thoracic centre after trial evaluation of the device for a three month period.

## Materials and methods

A pilot evaluation was performed in a regional thoracic unit over a period of two months to evaluate the utility of the device in our thoracic practice.

### Device

**Figure 1:**
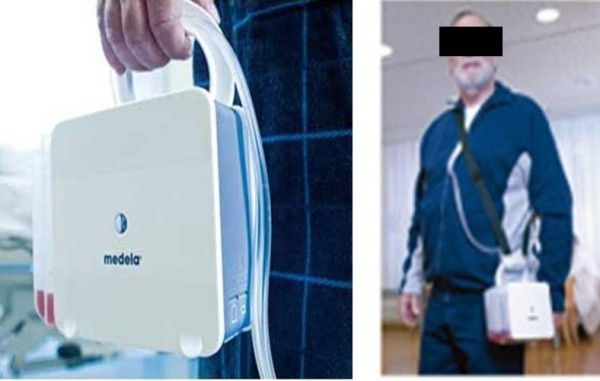
**Thopaz Chest Drain System**.

**Figure 2:**
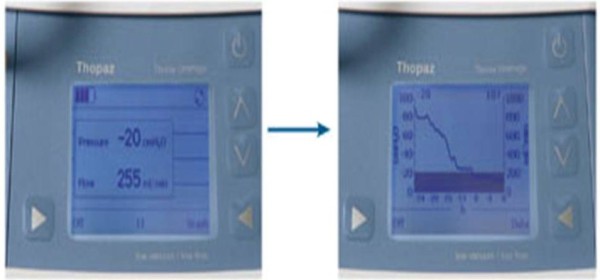
**Digital display and graphs of air-leak**.

Thopaz chest drain system is a portable suction unit which allows mobilization of the patient (Figures 1). This offers patients the benefit of suction and enables early mobility. It has scientific digital flow recordings with an in built alarm system (Figure 2). There are various alarms which alert the nurses regarding blocks, high volumes and battery status. The device also flushes the collection tubing connected to the inter-costal drain preventing blockage of drains.

The clinicians can assess air-leak in a scientific and objective manner as the data can be reviewed in a graphic format. Drain removal is performed when there is minimal flow and the graphs are stable.

### Evaluation

15 devices were evaluated between August and October 2008 on elective thoracic surgical patients undergoing video-assisted thoracic surgical procedures and elective lung resections. Patients undergoing pneumonectomy and those undergoing decortication were excluded from the trial evaluation.

### User Feedback

The staff were trained about the use, assembly, alarm management and care of the device in pre arranged sessions. After a few weeks to enable familiarity and establish practice, the nursing staff were asked to fill a structured questionnaire addressing overall device assessment, device assembly, ease of management and satisfaction rating them on a scale 1-6 ( 1:Excellent, 2:Very good, 3:Good, 4:Satisfactory, 5: Needs improvement and 6 Poor). Patients with pneumothoraces who had chest drains and wall suction prior to surgery who had Thopaz following the operation were requested to give their feedback. The medical staff were requested to state their opinion regarding the device.

## Results

120 patients were evaluated over 2.5 months. The procedures were lobectomy, wedge resection, VATS lung biopsy, VATS pleural biopsy and VATS bullectomy pleurectomy.

15 staff nurses evaluated the device and made their assessments on the following factors: overall device assessment, device setup and completeness, alarm management and instructions for use.

The staff feedback evaluation rated the device as very good or good in most categories. The Median scores and range are as follows with the following figures illustrating the median scores.

Overall: 2 (2-3) Efficacy: 2 (2-3)

Vacuum adjustment: 2 (1-4) Flow Readings: 3 (1-5)

Display: 3 (2-4) Alarm System: 3 (1-5)

Setup: 2 (1-3) Canister Change: 2 (1-3)

### Instructions

**Figure 3:**
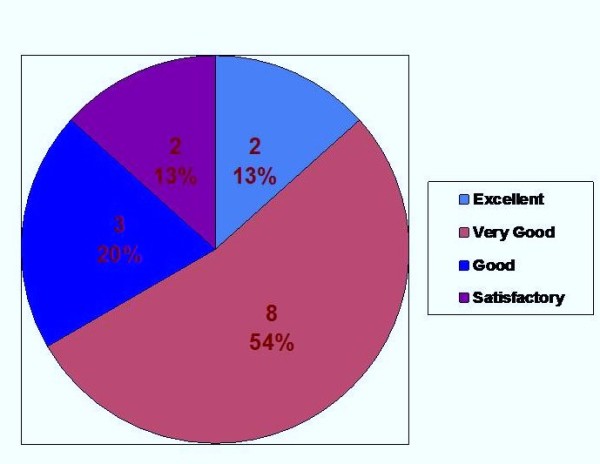
**Assessment on Instructions for use**.

The staff feedback on the instructions which accompanied the system including the error message and alarm management was rated as excellent by 13%, very good by 54%, good by 20% and satisfactory by 13% (Figure 3).

### Device Setup

The Staff were pleased that there was no need for priming which reduced the risk of spilling and infection risk as the canisters were sealed dry units. 4 rated the canister and canister setup as excellent with 7 and 4 rating them as very good and good respectively. The only negative feedback was the text marking on the canister which made it difficult to read in the night.

The tubing was rated as excellent by 6, very good by 6 and good by 3. The feature of constant flushing was perceived to be a positive attribute.

**Figure 4:**
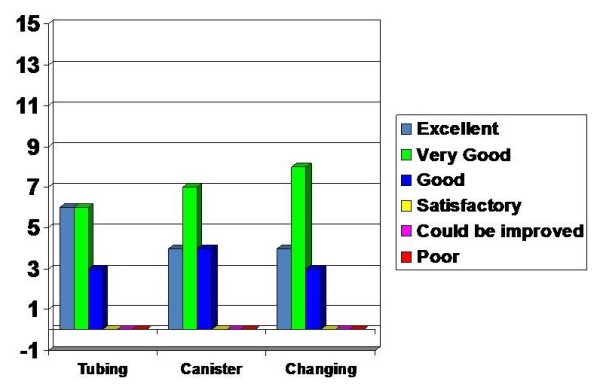
**Assessment on Setting up the device**.

Changing the canister and tubing was felt to be excellent by 4, very good by 8 and good by 3 staff (Figure 4).

### Device Characteristics

The assessments made on the device characteristics were assessed in various sections as vacuum setup and adjustment, flow rates and graphs, legibility of display and alarm system. The vacuum setup and adjustment was assessed to be excellent by 4, very good by 6, good by 4 and satisfactory by 1.

The flow rates and the graphical display were rated as follows: excellent (1), very good (5), good (5), satisfactory (1) and room for improvement (1). The legibility of display was felt to be very good by 5, good by 6, satisfactory by 3 and room for improvement by 1.

**Figure 5:**
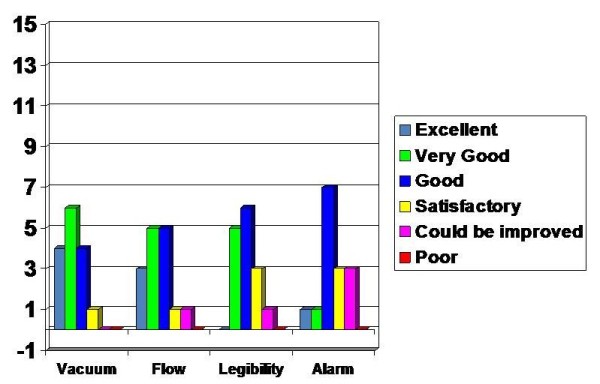
**Assessment on Device Specifics**.

The alarm warning and alarm system received mixed reviews as the feedback was influenced by the high sensitivity of the alarm systems. It was rated excellent (1), very good (1), good (7), satisfactory (3) and room for improvement (3) (Figure 5).

### Overall Device performance

The overall device performance had positive feedback with 13 rating it as very good and 2 rating it as good. The general utility of the device received similar assessment with 9 rating it as very good and 6 rating it as good.

**Figure 6:**
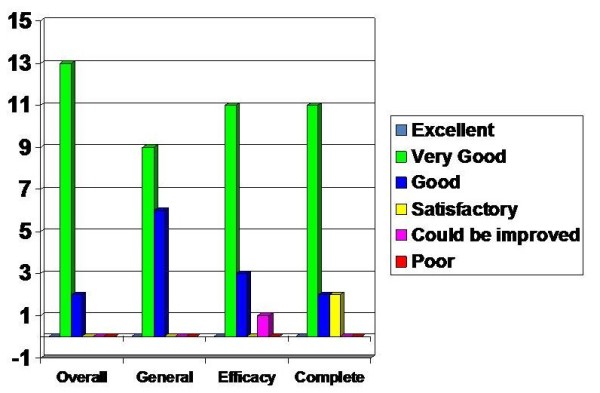
**Assessment on Overall device assessment**.

The device efficacy was felt to be very good by 11, good by 3 and satisfactory by 1 and similarly the overall device completeness was assessed as very good by 11, good by 2 and satisfactory by 2 (Figure 6).

Doctors' feedback stated that the drain management was more objective and scientific with the Thopaz.

Patients appreciated that it was portable and light which meant they could mobilise on suction giving them more independence. They preferred the lack of bubbling noise and the compactness over conventional drains and suction.

## Discussion

Chest drains are an integral part of thoracic surgery with patients having at least one drain after most thoracic surgical procedures. Closed chest drain systems have evolved from the time they were introduced in thoracic surgical practice. The three bottle underwater seal chest drainage has now progressed to single chamber devices with the liquid column acting as the one way valve to enable fluid and air to escape from the thoracic cavity.

Suction is applied to the chest drain systems in the presence of continuous air-leak or when the lung is not fully expanded [3,5,6]. There is a continuing debate on the role of suction in thoracic surgery [7,8]. Some surgeons practice includes continuous suction following pleurectomy and talc insufflations to enable adequate pleurodesis.

Currently suction is applied from the wall suction ports in the ward which are connected to the chest drain bottles. This has a logistical problem in wards without wall suction port. The flow in the wall suction ports can be variable and the suction delivered to the patient is influenced by a variety of factors like the length of the tubing from the wall suction port, the fluid in the chamber, the length of the tubing between the patient and drain bottle.

Thoracic surgical patients need to mobilise and have physiotherapy as a part of their post-operative care, constant suction can impede mobility. Continuous suction also poses privacy problems to young patients who have to stay in bed while having to use convenience facilities.

Patients sometimes are allowed to mobilise off suction and on suction when resting; this poses the potential infection risk as most patients tend to disconnect the tubing from the drain bottle, leave it on the floor and connect it back to the drain bottle.

There is an alternative option in patients with persistent air-leaks when the lungs are up which are not suction dependant. The drains can be connected to flutter-bags with Heimlich valves to enable mobilisation and even discharge home to remove the drains later [9]. There are various devises which measure digital air leak this has reduced inter-observer error in decision making in drain management in patients with air-leak [10].

In an ideal world the chest drainage system should be reliable, simple, safe, portable, cost efficient and offer objective real time data to help clinicians in the decision making of chest drain management. The device which we evaluated, the Thopaz portable chest drain and suction unit helps address most of these problems.

It enables the patients to mobilise, observing and recording the flows of the drainage through the patient in a digitally retrievable format. The manufacturers state the device provides suction only when required until the pleural pressure reaches the physiological limit. Hence the patient is not harmed by continuous suction. The Thopaz system has been shown to reduce the duration of chest drainage, delivers objective information in chest drain management, reduces the number of radiographs and is cost effective [11-13]. Our study focussed on the end users assessment of the device as it would be the users and patients who will be the proof of an effective system.

The Nurses were pleased that there was no need for priming which reduced the risk of spilling and infection risk as the canisters were sealed dry units. The onscreen graphs and alarms made management more safe which made air leak assessment accurate. The setting up was user friendly and the product could be cleaned with any alcohol group detergent.

The initial learning period had difficulties with alarm sensitivities, the difficulty with reading the letters on the canisters and some leakage from the canister during disposal. However these have been addressed in the newer version.

Patients appreciated that it was portable and light which meant they could mobilise on suction giving them more independence. They preferred the lack of bubbling noise and the compactness over conventional drains and suction. This comment was prominent in particularly the patients that were transferred with pneumothoraces on the standard under water seal bottle on continuous wall suction.

In our study the clinicians found the system to be user friendly and enabled effective decision making in the removal of chest drains. The perceived benefits also included utility in wards without wall suction, reduction in portable x-rays, decreased infection risk and better physiotherapy.

## Conclusion

Thopaz digital suction units were found to be user friendly and were liked by the staff and patients. The staff feedback stated the devices to be objective and scientific in making decisions about removal and enabled mobilisation.

## Competing interests

The authors declare that they have no competing interests.

## Authors' contributions

SR was involved with study design, collected the data, performed the data analysis and authored the manuscript, AB was involved in data collection and coauthored manuscript and TC collected data and co-authored manuscript. PBR is the principal investigator, devised the study and co authored the manuscript. All authors have read and approved the manuscript.
